# Editorial: Theory of mind in relation to other cognitive abilities, volume II

**DOI:** 10.3389/fpsyg.2025.1564067

**Published:** 2025-06-19

**Authors:** Ann Dowker, Douglas Frye

**Affiliations:** ^1^Department of Experimental Psychology, Oxford University, Oxford, United Kingdom; ^2^Department of Psychology, University of Pennsylvania, Philadelphia, PA, United States

**Keywords:** theory of mind, false belief (FB), child development, developmental disorders, autism, language, executive functions, central coherence

Theory of mind and its development have been the subject of much research over the last 40 years. It is generally thought to be very important in cognitive and social development. However, there is still much debate as to how it should be defined and even as to whether it constitutes a single entity. In particular, there is controversy as to the extent to which it should be seen as a specific cognitive module, or rather as dependent on, or mutually developing with, other cognitive abilities and characteristics, such as language, metacognition, executive function, and cognitive and perceptual styles that emphasize gist vs. detail (“strong” vs. “weak” central coherence). It is also possible that the theory of mind itself has several different components that may be related to different degrees, different cognitive abilities and characteristics. Any relations between the theory of mind and other cognitive characteristics may also vary with age, and may differ between typically developing children and those with autism and other atypical conditions.

Gaining a greater understanding of these issues is important for increasing our understanding of the theory of mind itself, the nature of cognitive development, the similarities and differences between typically and atypically developing children, and whether it is possible to devise interventions to improve the theory of mind, either directly or by improving other abilities. Between 2020 and 2023, we edited a Research Topic on the relation between theory of mind and other cognitive abilities. The current Research Topic is the second volume, and its goal is to extend those earlier findings by bringing together new articles on various aspects of the theory of mind and any concurrent and longitudinal relationships with other cognitive abilities and characteristics.

The articles in this Research Topic can be divided into three broad groups: the nature, correlates and predictors of theory of mind in children; the nature, correlates and predictors of theory of mind in adults; and the role of theory of mind difficulties in disorders.

With regard to theory of mind in children, Ünlüer investigated whether preschool (4- and 5-year-old) children's theory of mind skills and peer relationships predicted their subsequent school adjustment. There were indeed significant relationships. Theory of mind significantly predicted school adjustment as a whole positively predicting liking for school and negatively predicting school avoidance, while prosocial and aggressive behavior toward peers specifically predicted liking for school.

The other articles on children in this Research Topic look at characteristics that predict and may contribute to theory of mind, rather than those that follow on from it. Both articles suggest that certain language skills are important predictors.

de Villiers and de Villiers studied 258 children aged between 3 and 5 over a few months and tested them on three occasions on false belief reasoning and on the possible contributing factors of general language development, complement syntax, vocabulary, and inhibitory control. Cross-sectional and longitudinal regressions showed that all these factors contributed significantly to false belief reasoning. However, by the third assessment, the major proximal contribution was the child's comprehension of syntactic complements. The authors concluded that, as suggested by their earlier training studies, complement syntax makes an important specific contribution to false belief reasoning, but that vocabulary and executive function skills also form pathways to it.

Li and Leung assessed the language skills, executive functions and first-order and second-order false belief reasoning in 150 Mandarin-speaking preschoolers and early primary school children. They found that language was a significant independent predictor of both first-order and second-order false belief reasoning. Executive function predicted first-order false belief reasoning after controlling for age, but not after also controlling for language skills. However, it did continue to be a significant independent predictor of second-order theory of mind even after controlling for both age and language skills.

With regard to theory of mind in adults, one study, similar to Li and Leung's research with children, looked at the possible predictive roles of language and executive function.

Montgomery et al. investigated the theory of mind in adults along with its possible relation to language and executive function. The authors administered a series of advanced theory of mind tasks and tests of vocabulary and executive function to 207 adults. They found that the Strange Stories, Higher-Order False Belief, and Frith-Happé Animation tasks, though relatively weakly correlated, all loaded onto a common factor (?), which they considered to involve perspective-taking, within a narrative context, to represent a protagonist's mental state and use it to predict and explain their actions. This factor was more closely related to vocabulary than to executive function.

Mayrand et al. carried out a rather different type of study, looking at adults' interpretation of information communicated by gaze. They investigated how spatially dissociated vs. spatially combined effects of gaze (i.e., cases where an agent's inferred mental content implied by gaze is discrepant with the directional information communicated by gaze, vs. cases where the two types of cues provide concordant information) influence participants' target performance. They found that performance was worse when cue direction and mental content were discordant than when they were concordant. This effect was more marked when a social avatar served as a cue than when a comparison arrow was the cue. These findings suggest that a typical gaze communicates information about both what a person is attending to and the location of their attention.

Other studies examined the theory of mind in relation to disorders. The disorder that has been studied the most over the years with regard to associated limitations in theory of mind is autism spectrum disorder, as reflected in the articles in this Research Topic.

Qiao et al. conducted a comprehensive review of the literature from the past 30 years on the broader autism phenotype. First, they used the Web of Science Core Collection database to find articles on the autism phenotype in general published between 1994 and 2024. They then used the CiteSpace and VOS viewer software to further visualize and analyze the citations. They identified a total of 1,075 articles related to the broader autism phenotype. The annual number of publications on the subject has increased over the past 30 years. The largest number of publications came from the United States, followed by England and Canada. The United States also ranked first in terms of the extent to which its publications were cited.

Liu used photographs of social scenes to compare adolescents with autism spectrum disorder and controls on their ability to reason and make inferences about people's intentions. The adolescents with ASD performed significantly worse than the controls in making inferences about intentions. However, their ability to make physical causal inferences was unimpaired. Liu also investigated the relations between performance in these tasks and performance in tests of working memory and attention. Among individuals with ASD, working memory predicted physical causal inference, while divided attention predicted inferences about intention.

Gambra et al. investigated central coherence in typically-developing 6- to 11-year-olds and in several clinical groups: children with autism spectrum disorder; children with ADHD alone; children with ADHD and a non-verbal learning disability; and children with a social communication disorder. They used Gambra's (2020) Central Coherence Test to investigate children's use of context to make inferences and solve problems, rather than focusing predominantly on details. Children with ADHD alone did not differ from controls, but all the other clinical groups showed weaker central coherence than the controls. Those with ASD did not differ from the other clinical groups. Thus, autism was not the only condition associated with weak central coherence. The other disorders associated with weak central coherence in this study are also conditions that one might expect to be associated with theory of mind limitations, although this association was not explicitly tested in the present study.

Limitations in theory of mind have been found not only in patients with developmental disorders, but also in deaf children with hearing parents, possibly as a result of limited exposure to conversations about mental states. Wu et al. investigated whether deaf college students could be trained to improve their theory of mind. They trained 40 deaf students in theory of mind and compared them with 40 active controls, who received physical conversation training. The students, who were trained in theory of mind, improved significantly from pre-test to post-test on both cognitive and affective theory of mind tasks, and performed significantly better than the controls at post-test. These results suggest that training can improve theory of mind performance in deaf students.

The findings reported in the current Research Topic give rise to possibilities for further research. For example, to what extent and in what ways might the relations between the theory of mind and other cognitive abilities change with age? Do relations between the theory of mind and other cognitive abilities vary with culture and language? How do relations between the theory of mind and other cognitive abilities differ across different developmental disorders, and what implications might this have for intervention? The entire area of research is important and multifaceted and holds much promise for future development.
